# Supplementary material for: The GDAP1 p.Glu222Lys Variant-Weak Pathogenic Effect, Cumulative Effect of Weak Sequence Variants, or Synergy of Both Factors?
Source: Genes (Basel). 2022 Aug 27;13(9):1546. doi: 10.3390/genes13091546 (PMC9498914; doi:10.3390/genes13091546)
Supplement: Supplementary file 1 [file genes-13-01546-s001.zip › genes-1853659-supplementary.pdf]

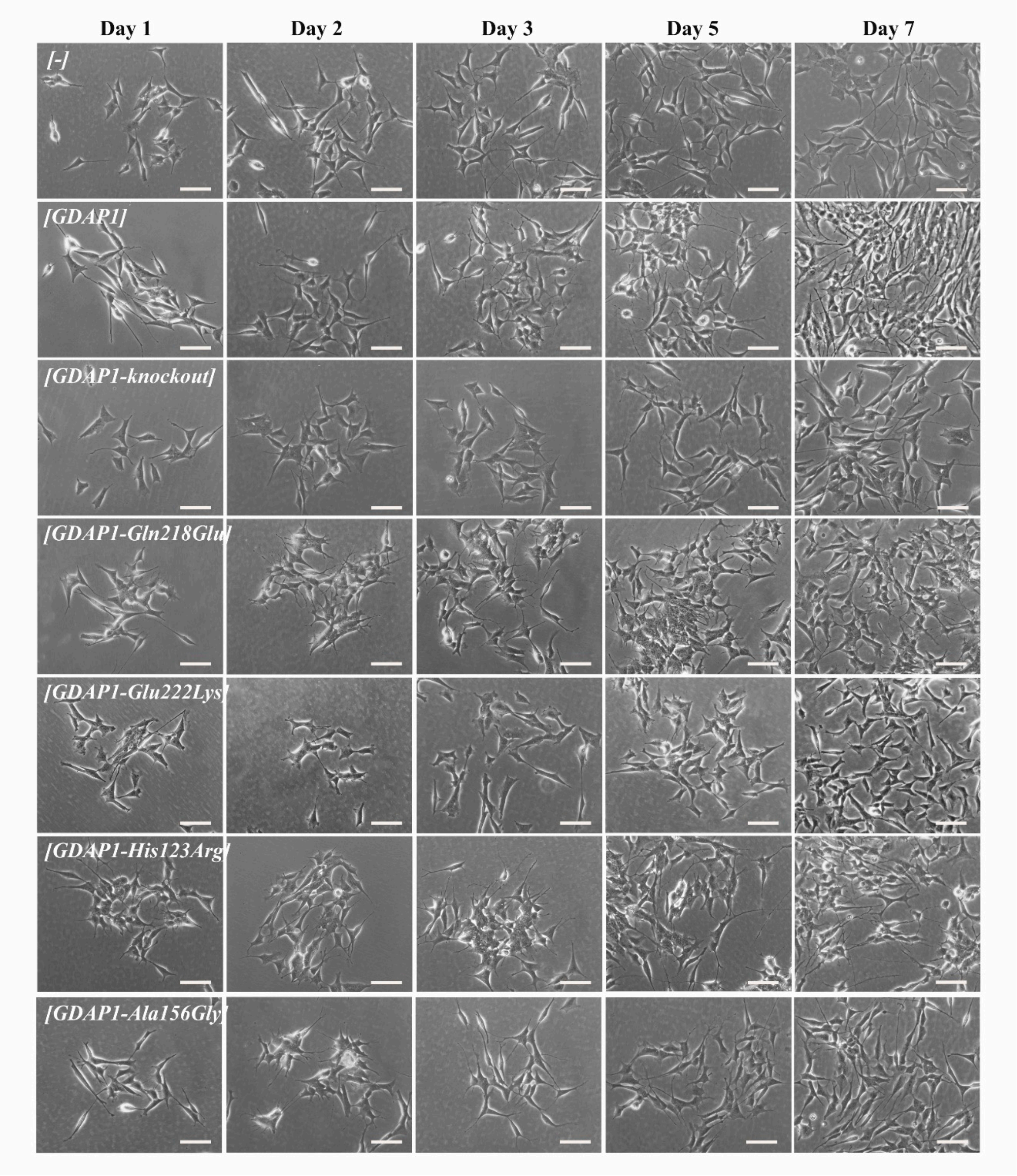

Figure S1. The expression of p.Glu222Lys results in a marked reduction of neurites like projection formation. The SH-SY5Y cells with knockout of GDAP1 or transfected with indicated GDAP1 alleles were visualized under light microscopy Zeiss Axio Imager at 24 (Day 1), 48 (Day 2), 72 (Day 3), 120 (Day 5) and 168 (Day 7) hours after cells seeding. Scale bar = 100 µm.

Table S1. List of the CMT genes analyzed in this study

AARS,	GDAP1,	PDK3,	VRK1,
ABHD12,	GJB1,	PEX1,	WNK1,
AIFM1,	GJB3,	PEX7,	YARS,
ARHGEF10,	GNB4,	PHYH,	MT-ATP6,
ARSA,	HADHB,	PLA2G6,	MT-ATP8,
ASAH,	HARS,	PLEKHG5,	MT-CO1,
ATL1,	HINT1,	PMM2,	MT-CO2,
ATL3,	HK1,	PMP22,	MT-CO3,
ATP7A,	HOXD10,	POLG,	MT-CYB,
BICD2,	HRNPA1	PRDM12,	MT-DLOOP,
BSCL2,	HSPB1,	PRNP,	MT-ND1,
C12ORF65,	HSPB3,	PRPS1,	MT-ND2,
CCT5,	HSPB8,	PRX,	MT-ND3,
CLTCL1,	IGHMBP2,	RAB7,	MT-ND4,
COX6A1,	IKBKAP,	RARS,	MT-ND5,
CTDP1,	INF2,	REEP1,	MT-ND6,
DARS,	IFRD1,	RFVT2,	MT-RYR1,
DCAF8,	KARS,	RNF170,	MT-RYR2,
DCTN1,	KCC3,	RYR1,	MT-TL1,
DHH,	SLC12A6,	SBF1,	MT-TM,
DHTKD1,	KIF1A/ATSV,	SBF2,	MT-TQ,
DNAJB2,	KIF1B,	SCN11A,	MT-TS1,
HSJ1,	KIF5A,	SCN9A,	MT-TS2,
DNAJC3,	LITAF,	SETX,	CARS2,
DNM2,	LMNA,	SH3TC2,	VARS2,
DNMT1,	LRSAM1,	SLC25A19,	ITPR1,
DRP2,	MARS,	SLC5A7,	ADCY6,
DST,	MED25,	SOX10,	SEPT9,
DYNC1H1,	MEN2B,	SPTLC1,	AIMP1,
EGR2,	MFN2,	SPTLC2,	DNAJB5,
FAM134B,	MORC2,	SPTLC3,	RARS2,
FBLN5,	MPZ,	SURF1,	FARSB,
FBXO38,	MTMR2,	TDP1,	NARS2,
FGD4,	MYH14,	TFG,	TARS,
FIG4,	NAGLU,	TRIM2,	PMP2,
FLVCR1,	NDRG1,	TRPV4,	HETX,
GALC,	NEFL,	TUBB3,	NOTCH3
GAN,	NGFB,	VABP,	
GARS,	NTRK1,	VCP,	
